# p38 MAPK Pathway in the Heart: New Insights in Health and Disease

**DOI:** 10.3390/ijms21197412

**Published:** 2020-10-08

**Authors:** Rafael Romero-Becerra, Ayelén M. Santamans, Cintia Folgueira, Guadalupe Sabio

**Affiliations:** Centro Nacional de Investigaciones Cardiovasculares (CNIC), 28029 Madrid, Spain; rafael.romero@cnic.es (R.R.-B.); ayelenmelina.santamans@cnic.es (A.M.S.); cintia.folgueira@cnic.es (C.F.)

**Keywords:** MAPK, p38, physiology, metabolism, signaling, hypoxia, arrhythmia

## Abstract

The p38 mitogen-activated kinase (MAPK) family controls cell adaptation to stress stimuli. p38 function has been studied in depth in relation to cardiac development and function. The first isoform demonstrated to play an important role in cardiac development was p38α; however, all p38 family members are now known to collaborate in different aspects of cardiomyocyte differentiation and growth. p38 family members have been proposed to have protective and deleterious actions in the stressed myocardium, with the outcome of their action in part dependent on the model system under study and the identity of the activated p38 family member. Most studies to date have been performed with inhibitors that are not isoform-specific, and, consequently, knowledge remains very limited about how the different p38s control cardiac physiology and respond to cardiac stress. In this review, we summarize the current understanding of the role of the p38 pathway in cardiac physiology and discuss recent advances in the field.

## 1. Introduction

p38α was identified by three groups in 1994 as a 38 kDa polypeptide that was phosphorylated after exposure to lipopolysaccharide (LPS), hyperosmolarity, or interleukin 1 (IL-1), that directly phosphorylates and activates the upstream kinase MAPKAP-K2 [1,2,3]. Later, three additional isoforms were described: p38β [4], p38γ (also called SAPK3 and ERK6) [5], and p38δ (also called SAPK4) [6]. The p38 family members are encoded by different genes, located tandemly in two chromosomes. The p38β (*Mapk11*) and p38γ (*Mapk12*) genes are located together on one chromosome (15 in mice and 22 in humans), whereas p38δ (*Mapk13*) and p38α (*Mapk14*) genes are located on another (17 in mice and six in humans). *Mapk12* is proposed to have arisen from tandem duplication of *Mapk11*, and the *Mapk13-Mapk14* gene unit is thought to have originated in a segmental duplication of the *Mapk11-Mapk12* unit [7].

The p38 family can be subdivided into two subsets, with p38α and p38β in one group and p38γ and p38δ in the other. This classification is based partly on amino-acid sequence identity; p38α and p38β are 75% identical, whereas p38γ and p38δ are 62% and 61% identical to p38α, respectively, while sharing 70% sequence identity with each other. The two p38 subsets also differ in their susceptibility to inhibitors, with in vitro and in vivo assays demonstrating that only p38α and p38β are inhibited by pyridinyl imidazoles (SB202190 and SB203580). A third difference between the p38 subgroups is substrate selectivity, with p38γ sharing common substrates with p38δ, and p38α with p38β [8,9,10].

p38 activity is regulated by phosphorylation at the end of a cascade composed of a MAPK kinase (MKK) and an MKK kinase (MEKK) [11,12,13]. The cascade is initiated by one of several MKK-phosphorylating MAP3Ks in cell-type- and stimulus-dependent manner. These MAP3Ks include mixed-lineage kinases (MLK), TGF β-activated kinase 1 (TAK1), MAPK/ERK kinase kinases (MEKK), TAO1 and TAO2, and apoptosis signal-regulating kinase-1 (ASK1) [14]. The p38s are activated by MKK-mediated dual phosphorylation of tyrosine and threonine residues in the conserved Thr–Xaa–Tyr motif (in p38, kinases Xaa is glycine, whereas in JNKs, it is proline in ERKs glutamic acid) [14]. Phosphorylation by MKKs is highly selective due to the specificity of the phosphorylation motif and the interaction of the MKK N-terminal region with different docking sites on the p38s. In addition, in T cells, p38 is activated by autophosphorylation [15] and also through AMPK-TAB1 [16], an alternative pathway that has been shown also in adipose tissue [17].

p38 activation is further tightly regulated by a group of inactivating phosphatases [11,14]. All p38 family members are widely expressed and considered ubiquitous, although p38β is most abundantly expressed in brain and adipose tissue, p38γ in skeletal muscle, and p38δ in secretory glands [5,6,18,19]. While all four p38s are expressed in the heart, the predominant family members in cardiomyocytes are p38α and p38γ. Extensive research into cardiac p38 function has suggested both protective and deleterious roles in the stressed myocardium. Which outcome predominates seems to depend in part on the model system under study and on the identity of the activated p38 family member. However, understanding remains limited of how the different p38 family members control cardiac physiology and respond to cardiac stress. In this review, we summarize current knowledge of p38 function in the heart and discuss recent advances.

## 2. Cardiovascular Development

In 2000, three groups independently showed that p38α is essential for normal cardiovascular development. Allen M. et al. demonstrated that genetic disruption of the p38α gene *Mapk14* was embryonically lethal [20]. Four months later, Adams R. et al. confirmed the essential requirement for p38α during early mouse development, showing that p38α deletion correlated with a massive reduction in myocardium formation and the appearance of blood-vessel malformations in the head region [21]. These authors suggested that p38α is necessary for placental organogenesis but is not necessary for other aspects of mammalian embryonic development [21]. Mudgett, J. et al. showed that p38α is required for the vascular remodeling associated with placental angiogenesis and trophoblast development [22].

Although p38 has been shown to play a key role in skeletal muscle development [23], less attention has been paid to its role in cardiac development. Several in vitro studies point to a possible role of p38 in cardiac development. For example, p38α activity is required for cardiomyocyte differentiation of P19CL6 cells, which is mediated via the activation of the transcription factor AP-1 [24]. p38α has also been shown to promote cardiogenesis over neurogenesis in ES cells [25]. Unfortunately, despite the strong suggestion of a cardiogenic role of p38α from cell-culture studies, in vivo data supporting this hypothesis are scarce. While embryos lacking p38α die due to defects in placental angiogenesis, cardiac-specific deletion of p38α results in normal development of the heart [26].

Several studies have shown that p38 kinases play an important role in different aspects of cardiogenesis, such as the regulation of cardiomyocyte differentiation and apoptosis. The role of p38 in cardiomyocyte differentiation was first suggested by studies using a specific inhibitor of p38α and p38β (SB203580), which demonstrated that p38 activity regulates important mitotic genes in cardiomyocytes. Neonatal mice lacking p38α have increased cardiomyocyte mitosis, suggesting that p38α acts as a negative regulator of cardiomyocyte proliferation ^2^. In adult cardiomyocytes, SB203580 and fibroblast growth factor 1 (FGF1) act synergistically to induce the expression of genes involved in proliferation and regeneration [27,28], indicating that the combination of FGF1 stimulation and p38α inhibition might rescue cardiac structure and function after injury [28]. The importance of p38 in cardiac differentiation was evident by the finding that p38α inhibition or gene deletion were sufficient to block cardiomyogenesis, suggesting that p38α activation constitutes an early switch in embryonic stem cell commitment to cardiomyogenesis [25]. The deletion or inhibition of p38α reduces expression of myocyte enhancer factor 2C (MEF2C), an important transcription factor acting on many genes encoding cardiac structural proteins [29]. The inhibition of p38 correlates with decreases in other cardiac transcription factors and MEF2C targets, such as atrial natriuretic factor (ANF) and myocardin, all of which contribute to the proper activation of the cardiac differentiation program during the early stages of development. p38α also regulates sarcomere assembly through the phosphorylation of ventricular myosin light chain 2 (MLC-2v), as well as the accumulation of α-actinin and its incorporation into sarcomeric units [29]. The lack of MEF2C activation upon p38 inhibition suppresses the expression of bone morphogenetic protein 2 (BMP-2), a key regulator of early cardiac cell development [30]. Most studies of cardiovascular development have focused on p38α; however, it is important to also define the role of other p38 family members in cardiac development. Mice with combined deletion of p38α and p38β display diverse developmental defects at mid-gestation, together with major cardiovascular abnormalities [31]. Embryos that express p38β only under the control of the p38α promoter display a similar heart phenotype as the double-knockout embryos, suggesting that heart development requires endogenous p38β expression [31]. Moreover, p38α and p38β have synergistic roles and specific functions in the regulation of cardiac gene expression during development, suggesting that some specific functions could be explained by differences in expression patterns [31]. It has been demonstrated the selective activation of p38 in the right ventricle during neonatal development and simultaneous inactivation in the left ventricle in neonatal mouse heart [32]. Cardiac-specific deletion of p38α and p38β in mice showed an abnormal gross morphology of the heart, developed right ventricle-specific enlargement dilation and, in consequence, a significant increase in cardiomyocyte proliferation, hypertrophy and a reduction in apoptosis without changes in the left ventricle. Furthermore, p38 inactivation induces XBP1 activity via IREα in the regulation of neonatal cardiomyocyte proliferation [32]. Finally, the role of p38γ and p38δ in cardiomyocyte development have not been assessed; however, animals lacking these kinases have smaller hearts at birth while conserving a normal number of cardiomyocytes, suggesting that these kinases contribute to the control of cardiomyocyte hypertrophy [33].

## 3. Cardiac Hypertrophy

The role of p38 kinases in cardiac hypertrophy was first suggested by the hypertrophic responses induced upon overexpression in cardiomyocytes of active forms of the upstream activators MKK3 and MMK6 [34,35,36]. The p38 pathway is also activated in cardiomyocytes exposed to hypertrophic stimuli, and hypertrophic growth is blocked by the SB203580-mediated inhibition of p38α/β [35]. However, MKK3 overexpression in cardiomyocytes also increased apoptosis [34]. The differences between the effects of MKK3 and MKK6 might point to distinct roles of p38 family members, a possibility supported by the finding that p38β activation in cultured cardiomyocytes induces characteristic features of hypertrophy [36], whereas p38α activation promotes cardiomyocyte apoptosis [37]. Consistent with this result, cardiac-specific knockout revealed a critical role for p38α in the cardiomyocyte survival pathway triggered by pressure overload, whereas hypertrophic growth was unaffected [37]. Although some groups have reported a role of p38α in the regulation of cardiac hypertrophy, most of those studies were based on the non-physiological overexpression of dominant-negative p38α or indirect strategies that might alter the function of other p38 family members [38,39].

The cardiomyocyte expression and subcellular localization of p38 isoforms was characterized by Dharmendra Dingar et al. in physiological conditions and in response to chronic pressure overload induced by transverse aorta constriction (TAC) [40]. TAC did not induce changes in the amount of p38α mRNA, whereas p38β and p38δ mRNA increased within 1 day of pressure overload and remained, while p38γ mRNA increased initially before returning to baseline levels by day 7. Despite these increases in mRNA abundance, the overall protein levels of p38β, p38γ, and p38δ were unaltered [40]. Confocal immunofluorescence analysis detected p38α and p38γ in the cytoplasm and nucleus at baseline; however, after chronic pressure overload, p38γ accumulated in the nucleus, whereas p38α distribution was unaffected. These localization differences would result in access to different substrates, and hence distinct functional effects [40].

The lack of pharmacological inhibitors of p38γ and p38δ has limited the study of these family members, though they were recently shown to control physiological and pathological cardiomyocyte growth. p38γ and p38δ are activated by angiotensin II and phosphorylate the mTOR inhibitor DEPTOR, inducing its degradation by the proteasome. Once DEPTOR is degraded, mTOR is released and activated, triggering protein synthesis and cardiomyocyte growth [33]. Mice lacking p38γ and p38δ have smaller hearts than controls and a reduced cardiomyocyte area [33]. Reduced hypertrophy capacity in these mice resulted in partial protection against angiotensin treatment [33]. Further experiments are needed to assess the therapeutic effects of p38γ and p38δ modulation during pathological hypertrophy.

## 4. Cardiac Regeneration

The best model for studying the molecular mechanisms of cardiac regeneration is the zebrafish, in which injury induces a cardiomyocyte proliferation that can overcome scar formation, thus allowing cardiac muscle regeneration [41]. Little attention has been paid to the role of p38 in zebrafish cardiac regeneration. p38α activation negatively regulates the proliferation of adult zebrafish cardiomyocytes [42], as also occurs in mammals [27]. During heart regeneration in adult zebrafish, the induction of p38α activity blocks cardiomyocyte proliferation, suggesting that p38α activity must be switched off in order to trigger cardiomyocyte proliferation and myocardial regeneration [42].

**Figure 1:**
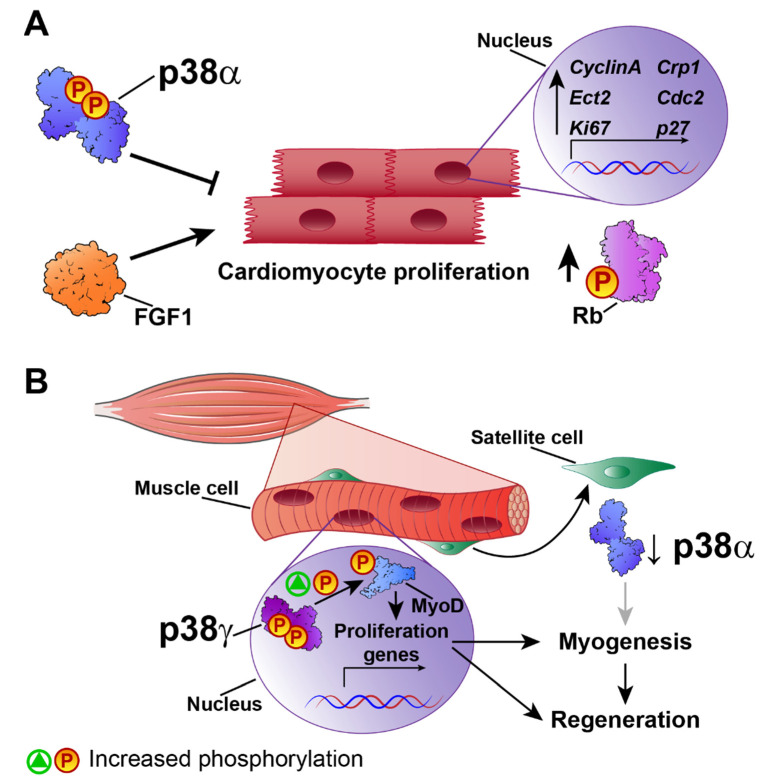
p38 in cardiovascular regeneration. **(A)** p38α blocks cardiovascular regeneration by inhibiting the expression of genes involved in cardiomyocyte proliferation and regeneration, such as *Ect2,* *Crp1, ki67, cdc2, cyclin A*, and p27, and reducing Rb phosphorylation to block cell-cycle progression. FGF1 stimulation has the opposite effect. **(B)** p38γ activates differentiation and myogenesis in satellite cells by phosphorylating MyoD and activating proliferation. p38α prevents p38γ activation in satellite cells, blocking regeneration. ↑ increase, ↓ decrease.

Mammalian hearts have a very low or non-existent regenerative capacity after cardiac injury. Nevertheless, in principle, signals that acutely trigger cardiomyocyte survival or modulate myoblast activity could be manipulated to promote cardiac regeneration and avoid heart failure. There is evidence implicating FGF1-upregulated genes in cardiac regeneration and cell-cycle control. The inhibition of p38 and stimulation of FGF1 act together to induce the expression of specific genes involved in proliferation and regeneration, such as cytokinesis regulator Ect2, cell-cycle-regulated protein 1 (CRP1), Ki67, cdc2, cyclin A, and the cell-cycle inhibitor p27 [27] (Figure 1A). Moreover, p38 inhibition and FGF1 induction lead to the phosphorylation of the key cell-cycle regulator Rb. These findings suggest that the promotion of cardiomyocyte proliferation by combined treatment with FGF1 and a p38 inhibitor could provide an alternative approach to the rescue of cardiac function after injury [28]. Studies of p38 isoforms in muscle regeneration have shown that p38β, p38γ, and p38δ are not required for efficient adult muscle regeneration and growth after injury [43], suggesting that p38α, in the absence of the other isoforms, especially p38γ, is sufficient to maintain satellite-cell-mediated myogenesis in vivo and in vitro [43].

The actions of p38 kinases in skeletal muscle regeneration have received much more attention than their roles in the heart. However, given the similarities between cardiac and skeletal muscle, the results of skeletal muscle studies can shed valuable light on the role of p38 in cardiac regeneration. Much research has focused on the potential role in adult myogenesis of p38γ, which is highly expressed in skeletal muscle. p38γ-deficient mice have a low muscle regeneration capacity after injury, with a reduced number of satellite cells that express myogenin prematurely and proliferate poorly. Mechanistically, p38γ phosphorylates MyoD, enhancing its occupancy of the myogenin promoter and thereby suppressing its expression. p38γ thus acts in opposition to p38α, blocking premature differentiation by inducing a repressive MyoD transcriptional complex during satellite-cell–mediated muscle growth and muscle regeneration [44] (Figure 1B). MyoD activates proliferation-associated genes but not differentiation genes, whose regulatory regions are repressed by ZEB1. Macrophages present in the injured muscles of Zeb1-deficient mice have low phosphorylation levels of p38, and forced p38 activation alleviates muscle damage and improves muscle regeneration [45]. In a later study, Brien P et al. demonstrated that a lack of p38α results in increased p38γ activation [46], suggesting that p38γ hyperactivation is involved in muscle regeneration.

Aging is characterized by a general decline in metabolic activity and function, with a loss of both skeletal and cardiac muscle accompanied by marked functional and structural impairment. Skeletal muscle has an outstanding regenerative capacity provided by its resident satellite cells. These normally quiescent cells are activated after injury to promote skeletal muscle regeneration [47]. Cardiomyocytes and cardiac muscle have long been thought to have lost these satellite cells and appear to lack the capacity for self-renewal and repair, preventing full recovery. Given the fundamental role of p38 in skeletal muscle regeneration, it is reasonable to postulate a causal link between cardiac muscle loss-of-function and p38.

To identify potential regeneration strategies based on satellite cells, a number of studies have investigated the effect of manipulating p38 signaling on aged satellite cells. For example, the manipulation of satellite cells enhances fibroblast growth factor receptor 1 (FGFR1) signaling and reduces p38α/β activation in satellite cells, increasing self-renewal and stimulating skeletal muscle regeneration [48]. Another study improved skeletal muscle repair and regeneration in rats using gold and gold-silver nanoparticles (AuNPs and Au-AgNPs, respectively) [49]. These nanoparticles regulate MyoD gene expression and activate p38α signaling, enhancing myoblast myogenic differentiation and promoting skeletal muscle regeneration. The effects of AuNPs and AuAgNPs were blocked with SB203580, suggesting that p38α is essential for myogenic differentiation [49]. These studies indicate an important role for p38 signaling in muscle regeneration; however, the underlying molecular mechanisms need further investigation to define the antagonic roles between p38α and p38γ. It will also be important to determine whether this antagonistic relationship operates in cardiac muscle.

## 5. p38 in Ischemia–Reperfusion Injury

Ischemic heart disease, the leading cause of death worldwide, is normally produced by a coronary artery occlusion that impairs cardiac blood flow. The ensuing decrease in oxygen delivery can lead to myocardial infarction. Reperfusion consists of the restoration of blood flow after the ischemia, and although this step is essential to avoid cardiomyocyte death, it also causes further damage associated with increased oxidative stress and inflammation [50,51]. Short ischemic episodes have been shown to protect the heart from a later ischemic insult, a process known as preconditioning or postconditioning, depending on whether it happens before or after the ischemia [52].

The p38 pathway is activated in response to ischemia–reperfusion and during preconditioning, producing a variety of results and cardiovascular scenarios [53]. In perfused rat hearts, p38 activation during ischemia and reperfusion is associated with a poor cardiac outcome. In contrast, during repetitive preconditioning treatments, p38 is maximally activated in the first episode, and activation gradually is reduced during sustained ischemia–reperfusion, improving cardiac functional recovery [53]. Interestingly, p38 activation is compartmentalized, with ischemia activating p38 in mitochondria, whereas during reperfusion p38 is activated in all cell compartments [54]. It would be interesting to determine whether different p38 family members are activated and localized in different cell compartments during these processes. Experiments performed in PC12 cells showed that moderate hypoxia (5% O_2_) increases p38γ and p38α phosphorylation, suggesting that ischemia might activate more than one p38 family member [55]. Most studies have examined p38α and β, both of which are inhibited by the widely used inhibitor SB203580. There is evidence to suggest that these two kinases have opposite roles, with p38α activation during ischemia triggering apoptosis, whereas p38β is responsible for pro-survival signaling during preconditioning [56]. Therefore, SB203580-mediated blockade of both isoforms during preconditioning results in loss of cardioprotective effects, whereas inhibition during I/R is beneficial [53].

The activation of p38 during preconditioning seems to be a consequence of adenosine release, which triggers the opening of ATP-sensitive potassium channels (KATP) during hypoxia [57,58]. The cardioprotective effects of p38 are thought to be due to phosphorylation of the downstream target Hsp27 and the subsequent enhancement of cytoskeletal stabilization during hypoxic stress [59]. In line with this idea, reactive oxygen species (ROS)-induced activation of p38α during hypoxia stabilizes hypoxia-inducible factor 1 (HIF-1) [60]. Moreover, the lack of dual specificity protein phosphatase 4 (DUSP4) leads to p38 hyper-phosphorylation and apoptosis [61]. Preclinical and clinical studies [62,63,64] with antioxidants highlight the importance of ROS as mediators of cardiac stress injury during I/R. ROS are potent p38 activators, but in vitro results with HL-1 cardiomyocytes also showed that p38 activation drives elevated ROS levels during ischemia–reperfusion [65]. In this analysis, the damaging effects of ROS were abolished by the p38 pan inhibitor BIRB796 [65]. Moreover, p38 inhibition with an antioxidant during ischemia–reperfusion is associated with improved cardiac recovery, decreased infarct size, and reduced apoptosis [66]. This was related to increased endogenous anti-oxidative enzyme activity and inhibition of oxidative stress [67]. The antioxidant Peroxiredoxin 1 and the ROS scavenger N-acetyl-l-cysteine have been also shown to decrease oxidative stress and block the activation of p38 and JNK, thus reducing apoptosis during ischemia–reperfusion [68]. Moreover, oxidative stress can directly regulate p38α activity and protein interactions by affecting the oxidation state of cysteines. Treatment with H_2_O_2_ induced p38-MKK3 disulfide dimer formation in isolated rat hearts and in an ischemia–reperfusion model, and dimer formation was abolished when the redox-sensitive cysteines were mutated or sterically inaccessible [69]. Further research is needed to determine whether p38 activation triggers ROS production, as proposed by Ashraf et al. [53,65], whether ROS induce p38 activation, or, more likely, there is reciprocal ROS–p38 regulation.

p38 has also been linked to cardiac inflammation through its promotion of the expression of regenerating islet-derived 3γ (Reg3γ), a protein associated with cardiac inflammatory signaling [70]. Moreover, treatment with the anti-inflammatory compound gamboge protects against infarction-induced inflammation by targeting the NF-κB–p38 pathway [71]. p38α activation is also associated with increased fibrosis during ischemia-induced cardiac remodeling [72]. Supporting this idea, inhibition of the p38 substrate MK2 impairs fibrotic scar formation after myocardial infarction [73]. The profibrotic effect of p38α was confirmed by conditional p38α deletion in myofibroblasts, which demonstrated that a lack of p38α blocks cardiac fibroblast differentiation into myofibroblasts, reducing fibrosis in response to ischemic injury [74]. p38 pathway activation by MKK6 overexpression results in interstitial and perivascular cardiac fibrosis [74], and p38 inhibition may underlie the beneficial effects of some statins on cardiac remodeling after myocardial infarction [75,76]. Supporting this idea, protease inhibitors induce cardioprotection in models of ischemia–reperfusion, in part by attenuating p38 phosphorylation, leading to reductions in injury, ROS levels, and infarct size [77].

The ischemia–reperfusion injury response is crucially determined by mitochondrial function and activity because mitochondria control cell metabolic status, intracellular calcium influx, oxidative stress, and apoptotic pathways, among other processes. p38 inhibition during ischemia–reperfusion decreases mitochondrial swelling, protects against ultra-structure alterations, and mitigates mitochondrial membrane depolarization [78]. There is also evidence that p38 activation during ischemia–reperfusion contributes to cardiac damage by triggering intracellular Ca^2+^ overload [79]. Pharmacological inhibition of p38 during ischemia–reperfusion induces cardioprotection by promoting phospholamban phosphorylation, increasing the activity of sarcoplasmic reticulum Ca^2+^-ATPase (SERCA2), and decreasing Ca^2+^ overload [80]. The role of p38 in the control of intracellular Ca^2+^ was corroborated in H9c2 cells: phosphodiesterase-inhibitor–mediated reduction in ERK1/2, JNK, and p38 activation reduced ischemia-induced apoptosis and restored normal calcium influx, oxidative stress levels, and eNOS expression [81]. p38 inhibition also potentiates the metformin-induced reduction in myocardial ischemia–reperfusion injury in non-obese type 2 diabetic rats [82].

**Figure 2:**
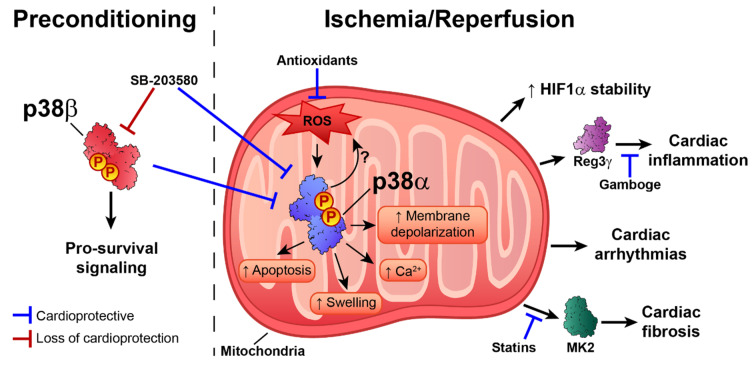
Dual role of p38 activation during preconditioning and ischemia–reperfusion injury. Activation of p38β during preconditioning triggers pro-survival signaling pathways, whereas decreased p38α activation during the ischemic episode leads to cardioprotection. On the other hand, ROS-induced p38α activation during the ischemic insult triggers HIF1-α stabilization; increases (↑) fibrosis, arrhythmias, and inflammation; and disrupts mitochondrial homeostasis. SB203580 administration during preconditioning increases myocardial injury, whereas administration during ischemia–reperfusion improves cardiac outcome. Indirect p38 downregulators, such as gamboge, statins, and antioxidants, seem to have beneficial effects when administered during or after the ischemia. Further research is needed to determine the precise reciprocity of ROS–p38 regulation. ↑ increase.

Ischemia–reperfusion injury also increases the risk of arrhythmia. p38 inhibition with SB203580 decreases ventricular tachycardia and ventricular fibrillation when administered to adult Wistar rats before or during ischemia, but not at the onset of reperfusion [83]. Mechanistically, this could be due to the ROS-dependent activation of p38 by ASK1 [84]. ASK1 would respond to the moderate increase in ROS during ischemia, but not to the higher levels of ROS observed in ischemia/reperfusion, acting as a redox sensor to mediate ROS-dependent signaling to p38 [84]. These findings highlight the importance of determining the optimal timing of p38 inhibition in order to achieve an efficient therapeutic response (Figure 2).

## 6. p38 in Heart Failure and Cardiac Arrhythmia

Heart failure (HF) is a major cardiac pathology and a global pandemic that affects more than 37 million people worldwide [85,86]. The p38 pathway is activated in HF, and specifically in the pathological cardiac remodeling that can lead to cardiac arrhythmia in the failing heart [87,88,89,90]. p38 plays an important role in the regulation of cardiac remodeling and cardiac contractility. Most studies suggest a negative role of p38 activation in extracellular matrix remodeling and the development of cardiac fibrosis, processes related to the development of HF [91,92,93]. Studies using transgenic animals with cardiac-specific expression of the activated p38 upstream kinases MKK3bE and MKK6bE showed that p38 pathway activation promotes cardiac interstitial fibrosis and increased expression of embryonic gene markers, similar to the expression profile observed in HF [89,94]. The profibrotic effect of p38 activation may be due to the induction in cardiomyocytes of TNF-α and IL-6 [89], which are closely associated with the development of fibrosis, adverse cardiac remodeling, and HF [95,96]. The effect of p38 activation on cardiac fibrosis is not limited to cardiomyocytes and also affects cardiac fibroblasts. The specific activation of the p38 pathway in cardiac fibroblasts leads to maladaptive cardiac remodeling with a profibrotic and hypertrophic phenotype and the activation of TGF-β signaling [97], a key cytokine involved in cardiac fibrosis and HF [95,96,98,99]. p38 is also necessary for the differentiation of fibroblasts into myofibroblasts, and specific deletion of p38α in cardiac fibroblasts or myofibroblasts reduces cardiac fibrosis in response to cardiac injury [74]. This is consistent with results showing that p38 inhibition decreases cardiac fibrosis and pro-inflammatory cytokine production [74,90], suggesting that p38 blockade is a possible treatment in HF. However, mice with cardiac-specific p38α deletion have a worse outcome to TAC-induced pressure overload, characterized by extensive cardiac fibrosis, dysfunction, and dilatation [37]. These opposite results might indicate that another family member is responsible for the protective effects of inhibitors or that p38 has opposite roles in cardiomyocytes versus other cardiac cells. Moreover, by promoting non-specific phosphorylations, overexpression of activated kinases may produce artificial cardiac structural and functional phenotypes. Further research is needed to determine the specific roles played by the different p38 family members in cardiac fibrosis and HF, since most studies have focused on p38α or the p38 pathway in general.

p38 can also control cardiomyocyte contractility, a predominant target of therapeutic strategies to treat HF [100]. The existing evidence indicates that p38 activation has an anti-inotropic effect in cardiac muscle [101,102,103,104,105,106]. Different mechanisms have been proposed for the negative effect of p38 on cardiac contractility. For example, p38 has been proposed to mediate the anti-inotropic effects of angiotensin II and ROS, which are also increased during HF, desensitizing the response of myofilaments to Ca^2+^ [102,105,107]. The mechanism underlying p38-mediated dampening of Ca^2+^ responsiveness is unknown, but two main possibilities have been proposed: modification of intracellular pH or phosphorylation of contractile proteins. However, studies have disproved the involvement of pH modification in myofilament sensitivity to Ca^2+^ [105,107], leaving phosphorylation of contractile proteins as the more likely mechanism. Analysis by Liao et al. did not detect increased p38-mediated troponin I phosphorylation, which is known to reduce myofilament responsiveness to Ca^2+^ [102]. Later work by Vahebi et al. showed that, rather than phosphorylation, p38 activation promotes the dephosphorylation of α-tropomyosin and troponin I. This was accompanied by a depression of cardiac and myofilament function and a decrease in maximum ATPase activity [106]. In agreement with this finding, p38 inhibition was found to promote troponin I phosphorylation [108]. p38-mediated dephosphorylation of α-tropomyosin and troponin I appears to be mediated by the protein phosphatases PP2C-α and PP2C-β, since p38, PP2C-α, and PP2C-β were found in the same protein complex in the sarcomere [106].

Another mechanism through which p38 might affect cardiac contractile function is the regulation of proteins involved in cardiomyocyte Ca^2+^ handling. During cardiac contraction, a depolarizing action potential promotes Ca^2+^ release from the sarcoplasmic reticulum, a process known as excitation-contraction coupling. Ca^2+^ enters the cell via L-type Ca^2+^ channels and, in much lower amounts, via the Na^+^/Ca^2+^ exchanger (NCX) [109]. This Ca^2+^ activates further sarcoplasmic reticulum Ca^2+^ release via the Ca^2+^-triggered Ca^2+^ release channel ryanodine receptor (RyR). The incoming cytosolic Ca^2+^ binds to the thin-filament protein troponin C, initiating myocyte contraction. For relaxation, calcium is removed from the cytosol by the action of Ca^2+^ transporters, mainly the sarcoplasmic/endoplasmic reticulum Ca^2+^-ATPase 2 (SERCA2) and sarcolemmal NCX [109]. Human HF is characterized by the reduced expression or activity of SERCA2 [110], resulting in increased diastolic Ca^2+^ concentrations due to defects in cytosolic Ca^2+^ removal, leading to reduced contractile force and impaired relaxation [110]. SERCA2 expression and activity are affected by p38 activation. In rat cardiomyocytes, p38 activation reduces SERCA2 mRNA expression and protein levels and reduces the activity of the SERCA2 gene promoter [111,112]. Scharf et al. showed that MK2/3 deletion results in increased protein levels and activity of SERCA2 in the heart and that SERCA2 gene expression is regulated by MK2-dependent Egr-1 transcription factor expression and promoter binding [113]. The p38 pathway regulates not only SERCA2 expression, but also its activity. For example, p38 inhibition increases the inotropic effect of endothelin-1 (ET-1) by modifying the SERCA2 inhibitory protein phospholamban (PLN). ET-1 treatment induces p38 phosphorylation [110,114] and promotes PLN phosphorylation at Ser-16 in the presence of p38 inhibition [80]. Similar results were obtained in a model of ischemia–reperfusion, which activated p38 and reduced SERCA2 expression and activity, as well as PLN phosphorylation, whereas these effects were partially reversed by p38 inhibition [113]. In MK2/3 double knockout mice, PLN phosphorylation is increased in the heart at the Ca^2+^/calmodulin-dependent protein kinase II (CaMKII) site (Thr-17) [113]. In the dephosphorylated state, PLN binds to and inhibits SERCA2. Phosphorylation at Ser-16 or Thr-17 relieves this inhibition, promoting Ca^2+^ reuptake to the sarcoplasmic reticulum and thus increasing cardiac contractility [115]. By promoting PLN dephosphorylation, p38 activation can therefore limit cardiac contractility. Although the main phosphatase responsible for PLN dephosphorylation is protein phosphatase 1 (PP1), PLN dephosphorylation can also be mediated by protein phosphatase 2A (PP2A) [116,117]. p38 activation has been reported to promote PP2A translocation and activation in myocytes [118], and p38 inhibition reduces PP2A-mediated dephosphorylation of PLN [108,118]. Furthermore, PP2A activation could be the mechanism by which p38 activation inhibits the β-adrenergic receptor-mediated contractile response in cardiomyoctes [104,118]. Kaikkonen et al. also proposed that decreased PP2A activity upon p38 inhibition could also promote PP1 inhibition, and that the p38 isoform responsible for these effects on SERCA2 regulation would likely be p38α [108]. Given that p38 also affects the activity of PP2C-α and PP2C-β, is feasible that p38 also affects the activity of PP2Ce, a novel member of the PP2C family that has been reported to be a specific and potent PLN phosphatase [116].

p38α also appears to participate in α-adrenergic-mediated *Ncx1* gene upregulation [119]. The overexpression of active MKK3 and MKK6 was sufficient to induce NCX1 upregulation in isolated cardiomyocytes, and this effect was mediated primarily by p38α [119,120]. Additionally, chemical inhibition of NCX1 promotes the formation of an NCX1-p38 complex and p38 activation. p38 activation induced by NCX1 inhibition has been suggested to be a physiological mechanism to compensate for loss of NCX1 activity by promoting *Ncx1* gene expression [121]. NCX1 activity and expression is also increased in heart failure [110] and the increased NCX1 activity increases Ca^2+^ extrusion to preserve the reduced diastolic Ca^2+^. This may compensate in part for the reduced SERCA2 function [110]; however, it can also promote other negative effects on cardiac contractility. For example, high NCX1 activity increases Ca^2+^ release from the cell, reducing sarcoplasmic reticulum Ca^2+^ stores and inducing contractile dysfunction. The elevated translocation of Ca^2+^ across the plasma membrane also results in a higher risk of delayed afterdepolarizations, which can cause arrhythmia and sudden death [110,122]. By reducing SERCA2 and increasing NCX1 function, p38 would seem to play an important role in the development of cardiac arrhythmia. Indeed, p38 activation has been linked to arrhythmogenic cardiac and ionic channel remodeling [83,87,88,123,124,125]. The pharmacological inhibition of p38 reduces the incidence of arrhythmia after ischemia–reperfusion by increasing the levels of phosphorylated connexin 43 (Cx43) [83]. Connexin clusters in the plasma membrane form gap junctions, which regulate cell-to-cell electrical and metabolic coupling and are essential for normal cardiac impulse transmission [126]. Cx43 is the most abundantly expressed connexin in cardiac myocytes, and its alteration has been linked to increased susceptibility to cardiac arrhythmia by altering action potential propagation in the heart [126,127]. There is strong evidence indicating a role for p38 in the regulation of Cx43. Several processes are involved in Cx43 regulation, including synthesis/degradation, phosphorylation/dephosphorylation at different residues, and cell membrane localization [128]. Cx43 expression is increased by p38 activation induced by several stimuli [129,130,131,132,133], but p38 is also implicated in Cx43 degradation [134]. The effect of p38 activation on Cx43 expression might depend on the activating stimulus and the cell type being studied. In cardiomyocytes, p38 activation appears to promote an increase in Cx43 mRNA and protein expression [129,130,131,132,133]. p38 can form a complex with Cx43 [135,136], and its activation has been reported to promote both Cx43 phosphorylation [135,137,138] and dephosphorylation (via PP2A activity) [136]. Adding further complexity, the increased phosphorylation of Cx43 upon p38 activation has been suggested to promote Cx43 degradation [137]. More research is needed to clarify the role of p38 activation in the regulation of Cx43 and gap junctions. However, despite the varying effects of p38 activation on Cx43 phosphorylation, the outcome of p38 activation is consistent across studies. p38-induced phosphorylation and dephosphorylation of Cx43 both lead to reduced cell-to-cell communication, impaired propagation of the action potential, and the development of cardiac arrhythmia [136,137,138]. Furthermore p38, inhibition improves cell-to-cell communication and reduces the incidence of arrhythmia [83,138].

**Figure 3:**
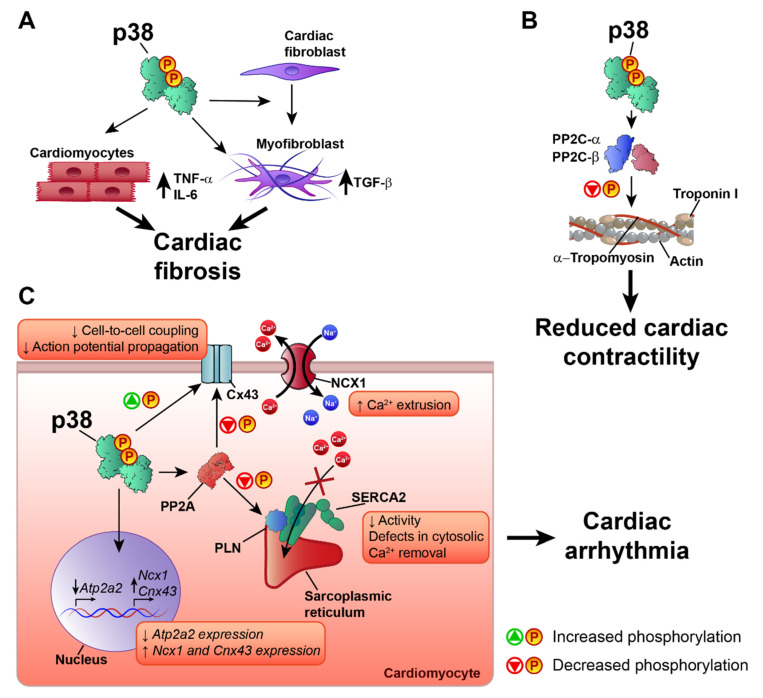
p38 in heart failure and cardiac arrhythmia. p38 activation participates in the development of heart failure and cardiac arrhythmia through three main mechanisms: **(A)** Increased cardiac fibrosis by induction of TNF-α and IL-6 in cardiomyocytes, differentiation of fibroblasts, and induction of TGF-β in cardiac myofibroblasts; **(B)** Reduced cardiac contractility due to dephosphorylation of a-tropomyosin and troponin I via PP2C-α/PP2C-β; **(C)** Promotion of cardiac arrhythmias due to reduced expression and activity of SERCA2 (*Atp2a2*), increased expression of NCX1 (*Ncx1*) and Cx43 (*Cnx43*), and altered Cx43 phosphorylation, inducing cardiac contractile dysfunction and altered action potential propagation. ↑ increase, ↓ decrease.

Many aspects of the role of p38 in the development of HF and cardiac arrhythmia remain to be clarified; however, most of the evidence points to a negative effect of p38 activation on the onset of HF and arrhythmias. The mechanisms involved include the development of cardiac fibrosis, alterations to Ca^2+^ handling proteins, and the modulation of gap junctions in the cardiomyocyte (Figure 3). Future research will need to address the role of the different p38 family members in these processes, since most studies have focused on p38α or p38α/β.

## 7. p38 Inhibitors in Clinical Trials

Despite the abundance of experimental evidence for the potential benefits of p38 inhibitors, clinical trials have failed to show improved cardiac outcomes after ischemia–reperfusion. The new anti-inflammatory medication losmapimod inhibits p38, and its administration to patients with non-ST-segment elevation myocardial infarction was well tolerated and improved the cardiac outcome [139]. However, in another study in acute myocardial infarction patients, losmapimod did not reduce the risk of major ischemic cardiovascular events, resulting in the withdrawal of the clinical trial [140].

Alternative approaches to p38 inhibition have been suggested in order to avoid undesirable side effects. The inhibition of MK2 in activated rheumatoid arthritis fibroblast-like synoviocytes avoided the modification of the secretion of chemokines like TNF-alpha, normally associated with the activation of other pro-inflammatory pathways like ERK and JNK during direct p38 inhibition [141]. Moreover, MK2^−/−^ mice showed improved ventricular recovery after ischemia–reperfusion, as well as reductions in infarct size and apoptosis [142]. MK2 inhibition with MMI-0100 after acute myocardial infarction inhibited cardiac fibrosis by enhancing primary cardiac fibroblast cell death while inhibiting cardiomyocyte apoptosis [73].

The lack of efficacy of p38 inhibitors in clinical trials might be due to the high similarity among p38 family members. The lack of isoform-specific inhibitors promotes the development of toxic secondary effects and probably leads to the triggering of regulatory feedback loops. Moreover, most studies used non-specific inhibitors, and very few studies have examined the specific p38 family member involved in a given action. Given the broad spectrum of undesired effects associated with the administration of inhibitors, the results obtained to date are hard to interpret. Further research with transgenic animal models will help to define the complex roles of p38 kinases.

## References

[1] Han J., Lee J.D., Bibbs L., Ulevitch R.J. (1994). A MAP kinase targeted by endotoxin and hyperosmolarity in mammalian cells. Science.

[2] Lee J.C., Laydon J.T., McDonnell P.C., Gallagher T.F., Kumar S., Green D., McNulty D., Blumenthal M.J., Keys J.R., Vatter S.W.L. (1994). A protein kinase involved in the regulation of inflammatory cytokine biosynthesis. Nature.

[3] Rouse J., Cohen P., Trigon S., Morange M., Alonso-Llamazares A., Zamanillo D., Hunt T., Nebreda A.R. (1994). A novel kinase cascade triggered by stress and heat shock that stimulates MAPKAP kinase-2 and phosphorylation of the small heat shock proteins. Cell.

[4] Jiang Y., Chen C., Li Z., Guo W., A Gegner J., Lin S., Han J. (1996). Characterization of the structure and function of a new mitogen-activated protein kinase (p38beta). J. Biol. Chem..

[5] Li Z., Jiang Y., Ulevitch R.J., Han J. (1996). The primary structure of p38 gamma: A new member of p38 group of MAP kinases. Biochem. Biophys. Res. Commun..

[6] Jiang Y., Gram H., Zhao M., New L., Gu J., Feng L., Di Padova F., Ulevitch R.J., Han J. (1997). Characterization of the structure and function of the fourth member of p38 group mitogen-activated protein kinases, p38delta. J. Biol. Chem..

[7] Li M., Liu J., Zhang C. (2011). Evolutionary history of the vertebrate mitogen activated protein kinases family. PLoS ONE.

[8] Hasegawa M., Cuenda A., Spillantini M.G., Thomas G.M., Buée-Scherrer V., Cohen P., Goedert M. (1999). Stress-activated protein kinase-3 interacts with the PDZ domain of alpha1-syntrophin. A mechanism for specific substrate recognition. J. Boil. Chem..

[9] Sabio G., Arthur J.S.C., Kuma Y., Peggie M., Carr J., Murray-Tait V., Centeno F., Goedert M., A Morrice N., Cuenda A. (2005). p38γ regulates the localisation of SAP97 in the cytoskeleton by modulating its interaction with GKAP. EMBO J..

[10] Sabio G., Reuver S., Feijoo C., Hasegawa M., Thomas G.M., Centeno F., Kuhlendahl S., Leal-Ortiz S., Goedert M., Garner C. (2004). Stress- and mitogen-induced phosphorylation of the synapse-associated protein SAP90/PSD-95 by activation of SAPK3/p38gamma and ERK1/ERK2. Biochem. J..

[11] Chang L., Karin M. (2001). Mammalian MAP kinase signalling cascades. Nat. Cell Biol..

[12] Cuadrado A., Nebreda A.R. (2010). Mechanisms and functions of p38 MAPK signalling. Biochem. J..

[13] Arthur J.S.C., Ley S.C. (2013). Mitogen-activated protein kinases in innate immunity. Nat. Rev. Immunol..

[14] Sabio G., Davis R.J. (2014). TNF and MAP kinase signalling pathways. Semin. Immunol..

[15] Salvador J.M., Mittelstadt P.R., Guszczynski T., Copeland T.D., Yamaguchi H., Appella E., Fornace A.J., Ashwell J.D. (2005). Alternative p38 activation pathway mediated by T cell receptor–proximal tyrosine kinases. Nat. Immunol..

[16] Lanna A., Henson S.M., Escors D., Akbar A.N. (2014). The kinase p38 activated by the metabolic regulator AMPK and scaffold TAB1 drives the senescence of human T cells. Nat. Immunol..

[17] Matesanz N., Bernardo E., Acín-Pérez R., Manieri E., Pérez-Sieira S., Hernández-Cosido L., Montalvo-Romeral V., Mora A., Rodriguez É., Leiva-Vega L. (2017). MKK6 controls T3-mediated browning of white adipose tissue. Nat. Commun..

[18] Ittner A., Block H., Reichel C.A., Varjosalo M., Gehart H., Sumara G., Gstaiger M., Krombach F., Zarbock A., Ricci R. (2012). Regulation of PTEN activity by p38δ-PKD1 signaling in neutrophils confers inflammatory responses in the lung. J. Exp. Med..

[19] Uhlén M., Fagerberg L., Hallström B.M., Lindskog C., Oksvold P., Mardinoglu A., Sivertsson Å., Kampf C., Sjöstedt E., Asplund A. (2015). Tissue-based map of the human proteome. Science.

[20] Allen M., Svensson L., Roach M., Hambor J., McNeish J., Gabel C.A. (2000). Deficiency of the Stress Kinase P38α Results in Embryonic Lethality. J. Exp. Med..

[21] Adams R.H., Porras A., Alonso G., Jones M., Vintersten K., Panelli S., Valladares A., Perez L., Klein R., Nebreda A.R. (2000). Essential role of p38alpha MAP kinase in placental but not embryonic cardiovascular development. Mol. Cell.

[22] Mudgett J.S., Ding J., Guh-Siesel L., Chartrain N.A., Yang L., Gopal S., Shen M.M. (2000). Essential role for p38alpha mitogen-activated protein kinase in placental angiogenesis. Proc. Natl. Acad. Sci. USA.

[23] Keren A., Tamir Y., Bengal E. (2006). The p38 MAPK signaling pathway: A major regulator of skeletal muscle development. Mol. Cell. Endocrinol..

[24] Eriksson M., Leppä S. (2002). Mitogen-activated Protein Kinases and Activator Protein 1 Are Required for Proliferation and Cardiomyocyte Differentiation of P19 Embryonal Carcinoma Cells. J. Biol. Chem..

[25] Aouadi M., Bost F., Caron L., Laurent K., Brustel Y.L.M., Binétruy B. (2006). p38 Mitogen-Activated Protein Kinase Activity Commits Embryonic Stem Cells to Either Neurogenesis or Cardiomyogenesis. STEM CELLS.

[26] Gerits N., Kostenko S., Moens U. (2007). In vivo functions of mitogen-activated protein kinases: Conclusions from knock-in and knock-out mice. Transgenic Res..

[27] Engel F.B., Schebesta M., Duong M.T., Lu G., Ren S., Madwed J.B., Jiang H., Wang Y., Keating M.T. (2005). p38 MAP kinase inhibition enables proliferation of adult mammalian cardiomyocytes. Genes Dev..

[28] Engel F.B., Hsieh P.C.H., Lee R.T., Keating M.T. (2006). FGF1/p38 MAP kinase inhibitor therapy induces cardiomyocyte mitosis, reduces scarring, and rescues function after myocardial infarction. Proc. Natl. Acad. Sci. USA.

[29] Hernández-Torres F., Martínez-Fernández S., Zuluaga S., Nebreda Á., Porras A., Aránega A.E., Navarro F. (2008). A role for p38α mitogen-activated protein kinase in embryonic cardiac differentiation. FEBS Lett..

[30] Wu J., Kubota J., Hirayama J., Nagai Y., Nishina S., Yokoi T., Asaoka Y., Seo J., Shimizu N., Kajiho H. (2010). p38 Mitogen-Activated Protein Kinase Controls a Switch Between Cardiomyocyte and Neuronal Commitment of Murine Embryonic Stem Cells by Activating Myocyte Enhancer Factor 2C-Dependent Bone Morphogenetic Protein 2 Transcription. Stem Cells Dev..

[31] Barrantes I.D.B., Coya J.M., Maina F., Arthur J.S.C., Nebreda A.R. (2011). Genetic analysis of specific and redundant roles for p38 and p38 MAPKs during mouse development. Proc. Natl. Acad. Sci. USA.

[32] Yokota T., Li J., Huang J., Xiong Z., Zhang Q., Chan T.W., Ding Y., Rau C.D., Sung K., Ren S. (2020). p38 mitogen-activated protein kinase regulates chamber specific perinatal growth in heart. J. Clin. Investig..

[33] González-Terán B., López J.A., Rodríguez E., Leiva L., Martínez-Martínez S., Bernal J.A., Jiménez-Borreguero L.J., Redondo J.M., Vazquez J., Sabio G. (2016). p38γ and δ promote heart hypertrophy by targeting the mTOR-inhibitory protein DEPTOR for degradation. Nat. Commun..

[34] Wang Y., Su B., Sah V.P., Brown J.H., Han J., Chien K.R. (1998). Cardiac Hypertrophy Induced by Mitogen-activated Protein Kinase Kinase 7, a Specific Activator for c-Jun NH2-terminal Kinase in Ventricular Muscle Cells. J. Boil. Chem..

[35] Zechner D., Thuerauf D.J., Hanford D.S., McDonough P.M., Glembotski C.C. (1997). A Role for the p38 Mitogen-activated Protein Kinase Pathway in Myocardial Cell Growth, Sarcomeric Organization, and Cardiac-specific Gene Expression. J. Cell Biol..

[36] Wang Y., Huang S., Sah V.P., Ross J., Brown J.H., Han J., Chien K.R. (1998). Cardiac Muscle Cell Hypertrophy and Apoptosis Induced by Distinct Members of the p38 Mitogen-activated Protein Kinase Family. J. Biol. Chem..

[37] Nishida K., Yamaguchi O., Hirotani S., Hikoso S., Higuchi Y., Watanabe T., Takeda T., Osuka S., Morita T., Kondoh G. (2004). p38α Mitogen-Activated Protein Kinase Plays a Critical Role in Cardiomyocyte Survival but Not in Cardiac Hypertrophic Growth in Response to Pressure Overload. Mol. Cell. Biol..

[38] Braz J.C., Bueno O.F., Liang Q., Wilkins B.J., Dai Y.-S., Parsons S., Braunwart J., Glascock B.J., Klevitsky R., Kimball T.F. (2003). Targeted inhibition of p38 MAPK promotes hypertrophic cardiomyopathy through upregulation of calcineurin-NFAT signaling. J. Clin. Investig..

[39] Lopez I.P., Cariolato L., Maric D., Gillet L., Abriel H., Diviani D. (2013). A-kinase anchoring protein Lbc coordinates a p38 activating signaling complex controlling compensatory cardiac hypertrophy. Mol. Cell Biol.

[40] Dingar D., Merlen C., A Grandy S., Gillis M.-A., Villeneuve L.R., Mamarbachi A.M., Fiset C., Allen B.G. (2010). Effect of pressure overload-induced hypertrophy on the expression and localization of p38 MAP kinase isoforms in the mouse heart. Cell. Signal..

[41] Poss K.D., Wilson L.G., Keating M.T. (2002). Heart Regeneration in Zebrafish. Science.

[42] Jopling C., Suñè G., Morera C., Belmonte J.C.I. (2012). p38α MAPK regulates myocardial regeneration in zebrafish. Cell Cycle.

[43] Ruiz-Bonilla V., Perdiguero E., Gresh L., Serrano A.L., Zamora M., Sousa-Victor P., Jardí M., Wagner E.F., Muñoz-Cánoves P. (2008). Efficient adult skeletal muscle regeneration in mice deficient in p38β, p38γ and p38δ MAP kinases. Cell Cycle.

[44] Gillespie M.A., Le Grand F., Scimè A., Kuang S., Von Maltzahn J., Seale V., Cuenda A., Ranish J.A., Rudnicki M.A. (2009). p38-γ–dependent gene silencing restricts entry into the myogenic differentiation program. J. Cell Biol..

[45] Siles L., Ninfali C., Cortés M., Darling D.S., Postigo A. (2019). ZEB1 protects skeletal muscle from damage and is required for its regeneration. Nat. Commun..

[46] Brien P., Pugazhendhi D., Woodhouse S., Oxley D., Pell J.M. (2013). p38α MAPK Regulates Adult Muscle Stem Cell Fate by Restricting Progenitor Proliferation During Postnatal Growth and Repair. STEM CELLS.

[47] Sousa-Victor P., García-Prat L., Serrano A.L., Perdiguero E., Muñoz-Cánoves P. (2015). Muscle stem cell aging: Regulation and rejuvenation. Trends Endocrinol. Metab..

[48] Bernet J.D., Doles J.D., Hall J.K., Tanaka K.K., Carter T.A., Olwin B.B. (2014). p38 MAPK signaling underlies a cell-autonomous loss of stem cell self-renewal in skeletal muscle of aged mice. Nat. Med..

[49] Ge J., Liu K., Niu W., Chen M., Wang M., Xue Y., Gao C., Ma P.X., Lei B. (2018). Gold and gold-silver alloy nanoparticles enhance the myogenic differentiation of myoblasts through p38 MAPK signaling pathway and promote in vivo skeletal muscle regeneration. Biomater..

[50] Jin J.-K., Blackwood E.A., Azizi K.M., Thuerauf N.J., Fahem A.G., Hofmann C., Kaufman R.J., Doroudgar S., Glembotski C.C. (2016). ATF6 Decreases Myocardial Ischemia/Reperfusion Damage and Links ER Stress and Oxidative Stress Signaling Pathways in the Heart. Circ. Res..

[51] Wallert M., Ziegler M., Wang X., Maluenda A., Xu X., Yap M.L., Witt R., Giles C., Kluge S., Hortmann M. (2019). α-Tocopherol preserves cardiac function by reducing oxidative stress and inflammation in ischemia/reperfusion injury. Redox Biol..

[52] Tibaut M., Mekis D., Petrovic D. (2017). Pathophysiology of Myocardial Infarction and Acute Management Strategies. Cardiovasc. Hematol. Agents Med. Chem..

[53] Marais E., Genade S., Huisamen B., Strijdom H., Moolman J., Lochner A. (2001). Activation of p38 MAPK Induced by a Multi-cycle Ischaemic Preconditioning Protocol is Associated with Attenuated p38 MAPK Activity During Sustained Ischaemia and Reperfusion. J. Mol. Cell. Cardiol..

[54] Ballard-Croft C., Kristo G., Yoshimura Y., Reid E., Keith B.J., Mentzer R.M., Lasley R.D. (2005). Acute adenosine preconditioning is mediated by p38 MAPK activation in discrete subcellular compartments. Am. J. Physiol. Circ. Physiol..

[55] Conrad P.W., Rust R.T., Han J., Millhorn D.E., Beitner-Johnson D. (1999). Selective Activation of p38α and p38γ by Hypoxia. J. Biol. Chem..

[56] Saurin A.T., Martin J.L., Heads R., Foley C., Mockridge J.W., Wright M.J., Wang Y., Marber M.S. (2000). The role of differential activation of p38-mitogen-activated protein kinase in preconditioned ventricular myocytes. FASEB J..

[57] Yoshimura Y., Kristo G., Keith B.J., Jahania S.A., Mentzer R.M., Lasley R.D. (2004). The p38 MAPK Inhibitor SB203580 Blocks Adenosine A1 Receptor-Induced Attenuation of In Vivo Myocardial Stunning. Cardiovasc. Drugs Ther..

[58] Zhao T.C., Hines D.S., Kukreja R.C. (2001). Adenosine-induced late preconditioning in mouse hearts: Role of p38 MAP kinase and mitochondrial KATP channels. Am. J. Physiol. Circ. Physiol..

[59] Dana A., Skarli M., Papakrivopoulou J., Yellon D.M. (2000). Adenosine A1Receptor Induced Delayed Preconditioning in Rabbits. Circ. Res..

[60] Emerling B.M., Platanias L.C., Black E., Nebreda A.R., Davis R.J., Chandel N.S. (2005). Mitochondrial Reactive Oxygen Species Activation of p38 Mitogen-Activated Protein Kinase Is Required for Hypoxia Signaling. Mol. Cell. Biol..

[61] Barajas-Espinosa A., Basye A., Angelos M.G., Chen C.-A. (2015). Modulation of p38 kinase by DUSP4 is important in regulating cardiovascular function under oxidative stress. Free. Radic. Boil. Med..

[62] Kurian G.A., Paddikkala J. (2010). N-acetylcysteine and magnesium improve biochemical abnormalities associated with myocardial ischaemic reperfusion in South Indian patients undergoing coronary artery bypass grafting: A comparative analysis. Singap. Med J..

[63] Kurian G.A., Paddikkala J. (2009). Administration of aqueous extract of Desmodium gangeticum (L) root protects rat heart against ischemic reperfusion injury induced oxidative stress. Indian J. Exp. Boil..

[64] Kurian G.A., Suryanarayanan S., Raman A., Padikkala J. (2010). Antioxidant effects of ethyl acetate extract of Desmodium gangeticum root on myocardial ischemia reperfusion injury in rat hearts. Chin. Med..

[65] Ashraf M.I., Ebner M., Wallner C., Haller M., Khalid S., Schwelberger H.G., Koziel K., Enthammer M., Hermann M., Sickinger S. (2014). A p38MAPK/MK2 signaling pathway leading to redox stress, cell death and ischemia/reperfusion injury. Cell Commun. Signal..

[66] Hu J., Li Z., Xu L.-T., Sun A.-J., Fu X.-Y., Zhang L., Jing L.-L., Lu A.-D., Dong Y.-F., Jia Z.-P. (2014). Protective Effect of Apigenin on Ischemia/Reperfusion Injury of the Isolated Rat Heart. Cardiovasc. Toxicol..

[67] Yang X., Yang J., Hu J., Li X., Zhang X., Li Z. (2015). Apigenin attenuates myocardial ischemia/reperfusion injury via the inactivation of p38 mitogen-activated protein kinase. Mol. Med. Rep..

[68] Guo W., Liu X., Li J., Shen Y., Zhou Z., Wang M., Xie Y., Feng X., Wang L., Wu X. (2018). Prdx1 alleviates cardiomyocyte apoptosis through ROS-activated MAPK pathway during myocardial ischemia/reperfusion injury. Int. J. Biol. Macromol..

[69] Bassi R., Burgoyne J.R., DeNicola G.F., Rudyk O., DeSantis V., Charles R.L., Eaton P., Marber M.S. (2017). Redox-dependent dimerization of p38α mitogen-activated protein kinase with mitogen-activated protein kinase kinase. J. Biol. Chem..

[70] Säkkinen H., Aro J., Kaikkonen L., Ohukainen P., Näpänkangas J., Tokola H., Ruskoaho H., Rysä J. (2016). Mitogen-activated protein kinase p38 target regenerating islet-derived 3γ expression is upregulated in cardiac inflammatory response in the rat heart. Physiol. Rep..

[71] Na D., Aijie H., Bo L., Zhilin M., Long Y. (2017). Gambogic acid exerts cardioprotective effects in a rat model of acute myocardial infarction through inhibition of inflammation, iNOS and NF-κB/p38 pathway. Exp. Ther. Med..

[72] Ahmad F., Tomar D., A C S.A., Elmoselhi A.B., Thomas M., Elrod J.W., Tilley D.G., Force T. (2020). Nicotinamide riboside kinase-2 alleviates ischemia-induced heart failure through P38 signaling. Biochim. et Biophys. Acta (BBA) - Mol. Basis Dis..

[73] Xu L., Yates C.C., Lockyer P., Xie L., Bevilacqua A., He J., Lander C., Patterson C., Willis M.S. (2014). MMI-0100 inhibits cardiac fibrosis in myocardial infarction by direct actions on cardiomyocytes and fibroblasts via MK2 inhibition. J. Mol. Cell. Cardiol..

[74] Molkentin J.D., Bugg D., Ghearing N., Dorn L.E., Kim P., Sargent M.A., Gunaje J., Otsu K., Davis J. (2017). Fibroblast-Specific Genetic Manipulation of p38 Mitogen-Activated Protein Kinase In Vivo Reveals Its Central Regulatory Role in Fibrosis. Circulation.

[75] Wang M., Li Z., Zhang X., Xie X., Zhang Y., Wang X., Hou Y. (2015). Rosuvastatin Attenuates Atrial Structural Remodelling in Rats with Myocardial Infarction through the Inhibition of the p38 MAPK Signalling Pathway. Hear. Lung Circ..

[76] Li M., Liu F., Sang M., Sun X., Li L., Wang X. (2018). Effects of atorvastatin on p38 phosphorylation and cardiac remodeling after myocardial infarction in rats. Exp. Ther. Med..

[77] Prompunt E., Sanit J., Barrère-Lemaire S., Nargeot J., Noordali H., Madhani M., Kumphune S. (2018). The cardioprotective effects of secretory leukocyte protease inhibitor against myocardial ischemia/reperfusion injury. Exp. Ther. Med..

[78] Kumphune S., Surinkaew S., Chattipakorn S.C., Chattipakorn N. (2015). Inhibition of p38 MAPK activation protects cardiac mitochondria from ischemia/reperfusion injury. Pharm. Biol..

[79] Song N., Ma J., Meng X.-W., Liu H., Wang H., Song S.-Y., Chen Q.-C., Liu H.-Y., Zhang J., Peng K. (2020). Heat Shock Protein 70 Protects the Heart from Ischemia/Reperfusion Injury through Inhibition of p38 MAPK Signaling. Oxidative Med. Cell. Longev..

[80] Zhu S., Xu T., Luo Y., Zhang Y., Xuan H., Ma Y., Pan D., Li N., Zhu H. (2017). Luteolin Enhances Sarcoplasmic Reticulum Ca2+-ATPase Activity through p38 MAPK Signaling thus Improving Rat Cardiac Function after Ischemia/Reperfusion. Cell. Physiol. Biochem..

[81] Lee M.-L., Sulistyowati E., Hsu J.-H., Huang B.-Y., Dai Z.-K., Wu B.-N., Chao Y.-Y., Yeh J.-L. (2019). KMUP-1 Ameliorates Ischemia-Induced Cardiomyocyte Apoptosis through the NO⁻cGMP⁻MAPK Signaling Pathways. Molecules.

[82] Sanit J., Prompunt E., Adulyaritthikul P., Nokkaew N., Mongkolpathumrat P., Kongpol K., Kijtawornrat A., Petchdee S., Kumphune S. (2019). Combination of metformin and p38 MAPK inhibitor, SB203580, reduced myocardial ischemia/reperfusion injury in non-obese type 2 diabetic Goto-Kakizaki rats. Exp. Ther. Med..

[83] Surinkaew S., Kumphune S., Chattipakorn S., Chattipakorn N. (2013). Inhibition of p38 MAPK During Ischemia, But Not Reperfusion, Effectively Attenuates Fatal Arrhythmia in Ischemia/Reperfusion Heart. J. Cardiovasc. Pharmacol..

[84] Meijles D.N., Cull J.J., Markou T., Cooper S.T., Haines Z.H., Fuller S.J., O’Gara P., Sheppard M., Harding S.E., Sugden P.H. (2020). Redox Regulation of Cardiac ASK1 (Apoptosis Signal-Regulating Kinase 1) Controls p38-MAPK (Mitogen-Activated Protein Kinase) and Orchestrates Cardiac Remodeling to Hypertension. Hypertension.

[85] Ziaeian B., Fonarow G.C. (2016). Epidemiology and aetiology of heart failure. Nat. Rev. Cardiol..

[86] Savarese G., Lund L.H. (2017). Global Public Health Burden of Heart Failure. Card. Fail. Rev..

[87] Cardin S., Li D., Thorin-Trescases N., Leung T.-K., Thorin E., Nattel S. (2003). Evolution of the atrial fibrillation substrate in experimental congestive heart failure: Angiotensin-dependent and -independent pathways. Cardiovasc. Res..

[88] Li D., Shinagawa K., Pang L., Leung T.K., Cardin S., Wang Z., Nattel S. (2001). Effects of angiotensin-converting enzyme inhibition on the development of the atrial fibrillation substrate in dogs with ventricular tachypacing-induced congestive heart failure. Circulation.

[89] Li M. (2005). p38 MAP Kinase Mediates Inflammatory Cytokine Induction in Cardiomyocytes and Extracellular Matrix Remodeling in Heart. Circulation.

[90] Kyoi S., Otani H., Matsuhisa S., Akita Y., Tatsumi K., Enoki C., Fujiwara H., Imamura H., Kamihata H., Iwasaka T. (2006). Opposing effect of p38 MAP kinase and JNK inhibitors on the development of heart failure in the cardiomyopathic hamster. Cardiovasc. Res..

[91] Khan R., Sheppard R.J.M.F. (2006). Fibrosis in heart disease: Understanding the role of transforming growth factor-β1 in cardiomyopathy, valvular disease and arrhythmia. Immunology.

[92] Gonzalez A., Schelbert E.B., Díez J., Butler J. (2018). Myocardial Interstitial Fibrosis in Heart Failure. J. Am. Coll. Cardiol..

[93] Segura A.M., Frazier O.H., Buja L.M. (2012). Fibrosis and heart failure. Hear. Fail. Rev..

[94] Liao P., Georgakopoulos D., Kovacs A., Zheng M., Lerner D., Pu H., Saffitz J., Chien K., Xiao R.-P., Kass D.A. (2001). The in vivo role of p38 MAP kinases in cardiac remodeling and restrictive cardiomyopathy. Proc. Natl. Acad. Sci. USA.

[95] Shirazi L.F., Bissett J., Romeo F., Mehta J.L. (2017). Role of Inflammation in Heart Failure. Curr. Atheroscler. Rep..

[96] Porter K.E., Turner N.A. (2009). Cardiac fibroblasts: At the heart of myocardial remodeling. Pharmacol. Ther..

[97] Li Y., Li Z., Zhang C., Li P., Wu Y., Wang C., Lau W.B., Ma X.L., Du J. (2017). Cardiac Fibroblast–Specific Activating Transcription Factor 3 Protects Against Heart Failure by Suppressing MAP2K3-p38 Signaling. Circulation.

[98] Bujak M., Frangogiannis N.G. (2007). The role of TGF-β signaling in myocardial infarction and cardiac remodeling. Cardiovasc. Res..

[99] Lijnen P., Petrov V., Fagard R. (2000). Induction of Cardiac Fibrosis by Transforming Growth Factor-β1. Mol. Genet. Metab..

[100] Dorn G.W., Molkentin J.D. (2004). Manipulating Cardiac Contractility in Heart Failure. Circulation.

[101] Chen Y., Rajashree R., Liu Q., Hofmann P. (2003). Acute p38 MAPK activation decreases force development in ventricular myocytes. Am. J. Physiol. Circ. Physiol..

[102] Liao P., Wang S.-Q., Wang S., Zheng M., Zheng M., Zhang S.-J., Cheng H., Wang Y., Xiao R.-P. (2002). p38 Mitogen-Activated Protein Kinase Mediates a Negative Inotropic Effect in Cardiac Myocytes. Circ. Res..

[103] Szokodi I., Kerkelä R., Kubin A.-M., Sármán B., Pikkarainen S., Kónyi A., Horváth I.G., Papp L., Tóth M., Skoumal R. (2008). Functionally Opposing Roles of Extracellular Signal-Regulated Kinase 1/2 and p38 Mitogen-Activated Protein Kinase in the Regulation of Cardiac Contractility. Circulation.

[104] Zheng M., Zhang S.-J., Zhu W.-Z., Ziman B., Kobilka B.K., Xiao R.-P. (2000). β2-Adrenergic Receptor-induced p38 MAPK Activation Is Mediated by Protein Kinase A Rather than by Gior Gβγ in Adult Mouse Cardiomyocytes. J. Boil. Chem..

[105] Palomeque J., Sapia L., Hajjar R.J., Mattiazzi A., Petroff M.V. (2006). Angiotensin II-induced negative inotropy in rat ventricular myocytes: Role of reactive oxygen species and p38 MAPK. Am. J. Physiol. Circ. Physiol..

[106] Vahebi S., Ota A., Li M., Warren C.M., De Tombe P.P., Wang Y., Solaro R.J. (2007). p38-MAPK Induced Dephosphorylation of α-Tropomyosin Is Associated With Depression of Myocardial Sarcomeric Tension and ATPase Activity. Circ. Res..

[107] Espejo M.S., Aiello E.A., Sepúlveda M., Petroff M.G.V., Aiello E.A., De Giusti V.C. (2017). The reduced myofilament responsiveness to calcium contributes to the negative force-frequency relationship in rat cardiomyocytes: Role of reactive oxygen species and p-38 map kinase. Pflügers Archiv. Eur. J. Physiol..

[108] Kaikkonen L., Magga J., Ronkainen V.-P., Koivisto E., Perjés Á., Chuprun J.K., Vinge L.E., Kilpiö T., Aro J., Ulvila J. (2014). p38α regulates SERCA2a function. J. Mol. Cell. Cardiol..

[109] Bers D.M. (2000). Calcium fluxes involved in control of cardiac myocyte contraction. Circ. Res..

[110] Luo M., Anderson M.E. (2013). Mechanisms of altered Ca²⁺ handling in heart failure. Circ. Res..

[111] Heidkamp M.C., Scully B.T., Vijayan K., Engman S.J., Szotek E.L., Samarel A.M. (2005). PYK2 regulates SERCA2 gene expression in neonatal rat ventricular myocytes. Am. J. Physiol. Physiol..

[112] Andrews C., Ho P.D., Dillmann W.H., Glembotski C.C., McDonough P.M. (2003). The MKK6-p38 MAPK pathway prolongs the cardiac contractile calcium transient, downregulates SERCA2, and activates NF-AT. Cardiovasc. Res..

[113] Scharf M., Neef S., Freund R., Geers-Knörr C., Franz-Wachtel M., Brandis A., Krone D., Schneider H., Groos S., Menon M.B. (2013). Mitogen-Activated Protein Kinase-Activated Protein Kinases 2 and 3 Regulate SERCA2a Expression and Fiber Type Composition To Modulate Skeletal Muscle and Cardiomyocyte Function. Mol. Cell. Biol..

[114] Clerk A., Michael A., Sugden P.H. (1998). Stimulation of the p38 Mitogen-activated Protein Kinase Pathway in Neonatal Rat Ventricular Myocytes by the G Protein–coupled Receptor Agonists, Endothelin-1 and Phenylephrine: A Role in Cardiac Myocyte Hypertrophy?. J. Cell Biol..

[115] Kranias E.G., Hajjar R.J. (2012). Modulation of Cardiac Contractility by the Phopholamban/SERCA2a Regulatome. Circ. Res..

[116] Akaike T., Du N., Lu G., Minamisawa S., Wang Y., Ruan H. (2017). A Sarcoplasmic Reticulum Localized Protein Phosphatase Regulates Phospholamban Phosphorylation and Promotes Ischemia Reperfusion Injury in the Heart. JACC. Basic Transl. Sci..

[117] Lei M., Wang X., Ke Y., Solaro R.J. (2015). Regulation of Ca2+ transient by PP2A in normal and failing heart. Front. Physiol..

[118] Liu Q., Hofmann P.A. (2003). Modulation of protein phosphatase 2a by adenosine A1 receptors in cardiomyocytes: Role for p38 MAPK. Am. J. Physiol. Circ. Physiol..

[119] Xu L., Kappler C., Menick D. (2005). The role of p38 in the regulation of Na?Ca exchanger expression in adult cardiomyocytes. J. Mol. Cell. Cardiol..

[120] Menick D.R., Renaud L., Buchholz A., Müller J.G., Zhou H., Kappler C.S., Kubalak S.W., Conway S.J., Xu L. (2007). Regulation of Ncx1 gene expression in the normal and hypertrophic heart. Ann. New York Acad. Sci..

[121] Xu L., Kappler C.S., Mani S.K., Shepherd N.R., Renaud L., Snider P., Conway S.J., Menick D.R. (2009). Chronic Administration of KB-R7943 Induces Up-regulation of Cardiac NCX1. J. Biol. Chem..

[122] Pogwizd S.M., Schlotthauer K., Li L., Yuan W., Bers D.M. (2001). Arrhythmogenesis and Contractile Dysfunction in Heart Failure. Circ. Res..

[123] Lu Y.-Y., Chen Y.-C., Kao Y.-H., Chen S.-A., Chen Y.-J. (2013). Extracellular matrix of collagen modulates arrhythmogenic activity of pulmonary veins through p38 MAPK activation. J. Mol. Cell. Cardiol..

[124] Cheng W., Zhu Y., Wang H. (2016). The MAPK pathway is involved in the regulation of rapid pacing-induced ionic channel remodeling in rat atrial myocytes. Mol. Med. Rep..

[125] Mollenhauer M., Friedrichs K., Lange M., Gesenberg J., Remane L., Kerkenpaß C., Krause J., Schneider J., Ravekes T., Maass M. (2017). Myeloperoxidase Mediates Postischemic Arrhythmogenic Ventricular Remodeling. Circ. Res..

[126] Desplantez T. (2017). Cardiac Cx43, Cx40 and Cx45 co-assembling: Involvement of connexins epitopes in formation of hemichannels and Gap junction channels. BMC Cell Biol..

[127] Marian A.J., Asatryan B., Wehrens X.H. (2020). Genetic basis and molecular biology of cardiac arrhythmias in cardiomyopathies. Cardiovasc. Res..

[128] Solan J.L., Lampe P.D. (2009). Connexin43 phosphorylation: Structural changes and biological effects. Biochem. J..

[129] Polontchouk L., Ebelt B., Jackels M., Dhein S. (2001). Chronic effects of endothelin-1 and angiotensin-II on gap junctions and intercellular communication in cardiac cells. FASEB J..

[130] Salameh A., Schneider P., Mühlberg K., Hagendorff A., Dhein S., Pfeiffer D. (2004). Chronic regulation of the expression of gap junction proteins connexin40, connexin43, and connexin45 in neonatal rat cardiomyocytes. Eur. J. Pharmacol..

[131] Inoue N., Ohkusa T., Nao T., Lee J.-K., Matsumoto T., Hisamatsu Y., Satoh T., Yano M., Yasui K., Kodama I. (2004). Rapid electrical stimulation of contraction modulates gap junction protein in neonatal rat cultured cardiomyocytes. J. Am. Coll. Cardiol..

[132] Yao J., Ke J., Zhou Z., Tan G., Yin Y., Liu M., Chen J., Wu W. (2019). Combination of HGF and IGF-1 promotes connexin 43 expression and improves ventricular arrhythmia after myocardial infarction through activating the MAPK/ERK and MAPK/p38 signaling pathways in a rat model. Cardiovasc. Diagn. Ther..

[133] Salameh A., Krautblatter S., Baeβler S., Karl S., Gomez D.R., Dhein S., Pfeiffer D., Baessler S. (2008). Signal Transduction and Transcriptional Control of Cardiac Connexin43 Up-Regulation after α1-Adrenoceptor Stimulation. J. Pharmacol. Exp. Ther..

[134] Wang C.-Y., Liu H.-J., Chen H.-J., Lin Y.-C., Wang H.-H., Hung T.-C., Yeh H.-I. (2011). AGE-BSA down-regulates endothelial connexin43 gap junctions. BMC Cell Biol..

[135] Schulz R., Gres P., Skyschally A., Duschin A., Belosjorow S., Konietzka I., Heusch G. (2003). Ischemic preconditioning preserves connexin 43 phosphorylation during sustained ischemia in pig hearts in vivo. FASEB J..

[136] Xue J., Yan X., Yang Y., Chen M., Wu L., Gou Z., Sun Z., Talabieke S., Zheng Y., Luo D. (2019). Connexin 43 dephosphorylation contributes to arrhythmias and cardiomyocyte apoptosis in ischemia/reperfusion hearts. Basic Res. Cardiol..

[137] Ogawa T., Hayashi T., Kyoizumi S., Kusunoki Y., Nakachi K., Macphee D.G., Trosko J.E., Kataoka K., Yorioka N. (2004). Anisomycin downregulates gap-junctional intercellular communication via the p38 MAP-kinase pathway. J. Cell Sci..

[138] Zhong C., Chang H., Wu Y., Zhou L., Wang Y., Wang M., Wu P., Qi Z., Zou J. (2018). Up-regulated Cx43 phosphorylation at Ser368 prolongs QRS duration in myocarditis. J. Cell. Mol. Med..

[139] Newby L.K., Marber M.S., Melloni C., Sarov-Blat L., Aberle L.H., E Aylward P., Cai G., De Winter R.J., Hamm C.W., Heitner J.F. (2014). Losmapimod, a novel p38 mitogen-activated protein kinase inhibitor, in non-ST-segment elevation myocardial infarction: A randomised phase 2 trial. Lancet.

[140] O’Donoghue M.L., Glaser R., Cavender M.A., Aylward P.E., Bonaca M.P., Budaj A., Davies R.Y., Dellborg M., Fox K.A.A., Gutierrez J.A.T. (2016). Effect of Losmapimod on Cardiovascular Outcomes in Patients Hospitalized With Acute Myocardial Infarction. JAMA.

[141] Dulos J., Wijnands F.P.G., Alebeek J.A.J.V.D.H.-V., Van Vugt M.J.H., Rullmann J.A.C., Schot J.-J.G., De Groot M.W.G.D.M., Wagenaars J.L., Os R.V.R.-V., Smets R.L. (2013). p38 inhibition and not MK2 inhibition enhances the secretion of chemokines from TNF-? activated rheumatoid arthritis fibroblast-like synoviocytes. Clin. Exp. Rheumatol..

[142] Shiroto K., Otani H., Yamamoto F., Huang C.-K., Maulik N., Das D.K. (2005). MK2 gene knockout mouse hearts carry anti-apoptotic signal and are resistant to ischemia reperfusion injury. J. Mol. Cell. Cardiol..

